# Supplementary material for: Voltage-controlled magnetoelectric devices for neuromorphic diffusion process
Source: Nat Commun. 2025 May 30;16:5022. doi: 10.1038/s41467-025-58932-x (PMC12125179; doi:10.1038/s41467-025-58932-x)
Supplement: Supplementary file 1 — Supplementary Information [file 41467_2025_58932_MOESM1_ESM.pdf]

Supplementary Information

Voltage-Controlled Magnetoelectric Devices for Neuromorphic Diffusion Process

Yang Cheng[1,*], Qingyuan Shu[1], Albert Lee[1], Haoran He[1], Ivy Zhu[2], Minzhang Chen[1], Renhe Chen[3], Zirui Wang[4], Hantao Zhang[5], Chih-Yao Wang[6], Shan-Yi Yang[6], Yu-Chen Hsin[6], Cheng-Yi Shih[6], Hsin-Han Lee[6], Ran Cheng[4,5], and Kang L. Wang[1,*]

[1]*Department of Electrical and Computer Engineering, and Department of Physics and Astronomy, University of California, Los Angeles, CA, USA*

[2]*Department of Physics, The Ohio State University, Columbus, OH, USA*

[3]*Department of Electrical and Computer Engineering, University of California, San Diego, CA, USA*

[4]*Department of Electrical and Computer Engineering, University of California, Riverside, CA, USA*

[5]*Department of Physics and Astronomy, University of California, Riverside, CA, USA*

[6]*Industrial Technology Research Institute, Taiwan*

Corresponding author. E-mail: *cheng991@g.ucla.edu, *wang@ee.ucla.edu

Supplementary Notes

Supplementary Note 1

Langevin dynamics and neural field theory. In neural science, the Langevin equation is related to stochastic neural field model[1], which is very natural because the spiky chains of neurons are substantially affected by noise, which is not always Gaussian noise[2]. Researchers have found that in some situations individual neurons are influenced by Poisson-like noise.

One approach to study the stochastic neural field model is to use the diffusion approximation of a Langevin equation[3]

$$dV = (-\frac{V}{\tau} + \mu)dt + \sigma dW(t) \tag{1}$$

Here V is the membrane potential, $W(t)$ is a Wiener process and $dW(t)$ is a Gaussian noise, $\mu(t)$ is the mean background synaptic input, and $\sigma(t)$ is the size of the membrane fluctuations. This Langevin equation has a corresponding Fokker-Planck equation, which has been used to study the numerical schemes to track the probability density of a population of synaptically-coupled spiking neurons. Since studying the behavior of a huge number of single neurons raises great complexity, the Fokker-Planck equation has been used to study the thermal dynamics theory of neural systems. It is worth noting that current stochastic neural field theory is only a phenomenological theory.

Supplementary Note 2

Quantify the thermal stability factor. We apply out-of-plane field switching probability method to estimate thermal stability factor (Δ) and effective perpendicular magnetic anisotropy field,

$$P_{SW}(H_z) = 1 - \exp\left(-\operatorname{erfc}\left(\sqrt{\Delta}\left(1 - \frac{H_z}{H_{keff}}\right)\right)\frac{\sqrt{\pi}H_{keff}}{2R\sqrt{\Delta}}\right) \quad (2)$$

The fitted thermal stability is $48k_BT$ while the effective anisotropy field is 1152 Oe. Within single spin approximation, the effective field corresponding to the thermal stability is lower than the fitted value, suggesting that the possible existence of a minimum energy path different from the coherent switching.

Supplementary Note 3

Determine VCMA coefficient. To determine the VCMA coefficient of the free layer experimentally, we need to obtain the PMA energy (K_u) under applied gate voltage. Since the PMA energy is obtained by integrating the enclosed area of the M-H curve $K_u = \mu_0 \int H_x dM_x$ of the free layer magnetization m_{free}, and the reference layer does not influence the VCMA coefficient of the free layer, we fabricate a half-MTJ stack in which the reference layer is in-plane, and measure the tunneling magnetoresistance under the various in-plane bias field till the free layer is brought to the in-plane direction. Using $G(\theta) = G_0 + \Delta G \sin\theta$, and $M_x = M_s \sin\theta$ where G is the tunneling conductance, G_0 is the conductance when the two spins are perpendicular, M_x is the in-plane component of $\boldsymbol{m}_{free}$, and M_s is the saturation magnetization of CoFeB, we can convert the tunneling magnetoresistance to the M_x to obtain the M-H curve, as shown in Supplementary Fig. 3. Finally, the VCMA coefficient is extracted from a linear fit of the PMA data under different applied voltage. The VCMA coefficient extracted in this work is lower than our previous results and other reported values, which can reach as high as 100 fJ/Vm[4-9]. We attribute this reduction to challenges in integrating the MTJ stack into the CMOS BEOL process on an 8-inch wafer, which likely affected the interfacial quality and uniformity of the oxide layer. Despite this, the measured

coefficient remains sufficient for achieving reliable voltage-controlled switching in our neuromorphic diffusion process, demonstrating the feasibility of our approach even with the current limitations.

Supplementary Note 4

Derivation of noise distribution. By definition $\varepsilon(i) = A_i - A_{i-1}$, and taking our 8-bit unit as an example, the probability of the difference between two consecutive MTJ random number is $P_{\varepsilon(i)=\frac{k}{64}} = \sum_{j=0}^{255} P\left(A_i = \frac{k+j}{64} \middle| A_{i-1} = \frac{j}{64}\right)$, where $k + j \in \{0,1,\cdots,255\}$. We can convert it to matrix form,

$$P_{\varepsilon(i)} = TMP_{A_{i-1}}, \tag{5}$$

where $P_{\varepsilon(i)} = \begin{pmatrix} P_{\varepsilon(i)=-\frac{255}{64}} \\ \cdot \\ \cdot \\ \cdot \\ P_{\varepsilon(i)=\frac{255}{64}} \end{pmatrix}$, $T = \begin{pmatrix} R_{A_{i-1}} & 0 & 0 & \cdots & 0 \\ 0 & R_{A_{i-1}} & 0 & \cdots & 0 \\ 0 & 0 & R_{A_{i-1}} & \cdots & 0 \\ \vdots & \vdots & \vdots & \ddots & \vdots \\ 0 & 0 & 0 & \cdots & R_{A_{i-1}} \end{pmatrix}$, $P_{A_{i-1}} = \begin{pmatrix} P_{A_{i-1}=0} \\ \cdot \\ \cdot \\ \cdot \\ P_{A_{i-1}=\frac{255}{64}} \end{pmatrix}$

and $R_{A_{i-1}} = \begin{pmatrix} P_{A_{i-1}=\frac{255}{64}} \\ \cdot \\ \cdot \\ \cdot \\ P_{A_{i-1}=0} \end{pmatrix}$, which is the reverse of $P_{A_{i-1}}$. T is a matrix with a dimension of 511 X 256. Since in a Markov process when $i \to \infty$, value of A_i is deterministic and uniformly distributed from 0 to $\frac{255}{64}$, we have

$$P_{\varepsilon(i)} = \frac{1}{256} \sum_{j=0}^{255} TM\, P_{A_{i-1}}(A_{i-1} = \tfrac{j}{64}), \tag{6}$$

where $P_{A_{i-1}}\left(A_{i-1}=\frac{j}{64}\right)=\begin{pmatrix}0\\ \vdots\\ 0\\ 1\\ 0\\ \vdots\\ 0\end{pmatrix}$. Only $P_{A_{i-1}=\frac{j}{64}}=1$, the rest numbers in the vector are 0. From Eq. (6), we know that the distribution of noise $P_{\varepsilon(i)}$ depends on the switching probabilities of each MTJ. Thus, carefully choosing the $P_{P\to AP}$ and $P_{AP\to P}$ of the MTJs can get the $P_{\varepsilon(i)}$ to our desired distribution.

To improve the fit to a Gaussian, we can increase the number of bits in one MeRAM unit. Another possible route is to take the average of the noise generated independently by multiple MeRAM units. For instance, denoting the probability mass function of noise generated by a MeRAM unit as p_{ε_k}, then the distribution of the average of n noise outputs will be the convolution of the individual probability of ε

$$p_{\varepsilon}=p_{\varepsilon_1}*p_{\varepsilon_2}*\cdots*p_{\varepsilon_m} \tag{7}$$

In consequence, the precision improves from N to $N+m(N-1)$, thereby making the fitting better to the targeting distribution, as shown in Supplementary Fig. 4a and b. Here, $N=2^{n+1}-1$ is the number of states accessible by the noise ε_i. On the other hand, selecting unsuitable combinations of gate voltages and pulse widths can result in the generated distribution diverging from the intended Gaussian distribution, as demonstrated in Supplementary Figure 4c.

Supplementary Note 5

Comparison to CMOS-based technology. The basic unit of MeRAM unit is a P-cell (Supplementary Fig. 6a). A P-cell includes 4 transistors to separate the read and write operation.

In comparation, the CMOS-based biased random bit generator includes a 96-bit linear-feedback shift register (LFSR) pseudo random number generator (PRNG) and a bias generation circuit for achieving the tunability of switching probability. The LFSR is a shift register with XOR mixing between stages and generates uniformly distributed random bits in each clock cycle. The 96-bit random bits are grouped into 32 3-bit groups (Supplementary Fig. 6b). Each group is then passed through a Bias generator, which takes the three random bits to form a biased one with 3-bit precision (e.g., can be configured to probabilities in steps of 0.5^3 or 12.5%). A full MeRAM unit consists of P-cells, timing control, readout sense amplifiers, and a subtraction circuit (Supplementary Fig. 6c). During generation operation, a write voltage is applied across the MTJ to begin the bit generation; while during the read operation, a read voltage with inverse polarity is applied across the MTJ to avoid disturbance, and a readout voltage *Vcell* is generated via resistance division between the MTJ and a resistor. The timing control generates the pulse width required for each P-Cell. The Sense Amplifiers, built via StrongARM comparators, convert the MTJ states to digital signals by comparing P-cell readout voltage with a reference. Finally, a subtractor is used to post-process the bits generated by the MTJ into a normal distribution. The reference CMOS-based normal random number generator includes a 32-bit LFSR-based PRNG and a Box-Muller transform block (Supplementary Fig. 6d). The random bits are concatenated into a 32-b random number LFSR[31:0] and sent to the Box-Muller transform block. When two random numbers are collected, the numbers are mapped through a Box-Muller transform, which translates the uniform variables into normal distributed ones. The two designs are written in Verilog and synthesized into CMOS circuitry via Synopsis Design Compiler using a 28 nm High-Performance Commercial Process design kit. The maximum throughput, area, and energy are reported (Supplementary Table 1 and 2). It turns out that the single P-cell saves 80% of energy and achieves 16 to 28 times higher

FOM than LFSR-based random bit generator (RBG) . The performance of 32-bit MeRAM unit saves even more energy and area in generating normal random numbers than CMOS-based normal random number generator.

Supplementary Note 6

Comparison to spin-transfer torque (STT) and spin-orbit torque (SOT) based devices.

In the past, unconventional computing utilizing MRAM technology has primarily centered around employing spin-transfer torque (STT) based devices, such as probabilistic bit (p-bit) and spin-torque nano-oscillator[10-12]. These devices hold significant promise, largely due to the advanced state of STT-MRAM technology. However, when compared to VCMA-based devices, STT-based technology faces issues such as high energy consumption, limited endurance, and reduced speed as a result of incubation delay[13]. In addition, the STT-MRAM based probability bit requires a very low energy barrier (low PMA), which significantly decreases the retention time. For SOT-MRAM, the required three terminal devices as well as an external field makes the design of the circuit difficult. Moreover, the initialization of the magnetization to in-plane[14] by SOT requires, making it energy inefficient. Additionally, one key advantage of the VC-MTJ lies in its tunability of the energy barrier, allowing our MeRAM to simultaneously serve as both a P-bit and a non-volatile memory in a single device. This contrasts with STT- or SOT-based MTJs used as P-bits, which require a low energy barrier (PMA) for achieving the needed functionality, making them unsuitable for non-volatile memory applications. Conversely, VCMA-based devices tend to consume less energy, possess a faster respond time and bit density, making it suitable for large-

scale applications[15]. A Comparison between MeRAM and other memory technologies[16] is shown in Supplementary Table 3.

Supplementary Note 7

Implementation of DDPM. In the standard diffusion process (As shown in Fig. 4), the pixel values of the training set are normalized to [-1, 1]. We add noise pixel by pixel and shift the pixel mean in 1000 steps sequentially

$$\mathbf{x}_t = \sqrt{1-\beta_t}\,\mathbf{x}_{t-1} + \sqrt{\beta_t}\,\boldsymbol{z}, \tag{8}$$

where $\mathbf{x}_t$ is pixel data at the tth step, and β_t is the variance of the noise which increases linearly with t from 1×10^{-4} to 0.02. $\boldsymbol{z}$ subjects to $\mathcal{N}(0, 1)$ in software-based diffusion model, and in our MeRAM-based diffusion process, we add noise using the generated noise set. In the reverse diffusion process, the goal is to learn $p_\theta(\mathbf{x}_{t-1}|\mathbf{x}_t)$ given $q(\mathbf{x}_{t-1}|\mathbf{x}_t)$. This can be simplified to learning the noise added to the images, which is then implemented using a U-Net[17]. The number of channels at each resolution is 64, 128, 256, and 512, respectively. We use self-attention blocks between the convolution blocks at the 3rd and 4th resolution with group normalization applied, where the group number used is 32. We train the model with 16,000 images using Adam optimizer with a learning rate of 2×10^{-4}. It takes about 2 minutes to train one epoch with the batch size of 64 on a ml.p3.2xlarge AWS Sagemaker instance.

In our demonstration of image generation tasks (As shown in Fig. 3), we use an 80×80 VC-MTJs to represent the image data. Each pixel corresponds to an individual MTJ, where dark blue dots indicate the P state (1) and light blue dots indicate the AP state (0). Instead of modifying the pixel intensity values as in the standard DDPM due to the limited number of devices, our hardware-

based approach uses the noise generated by the MeRAM array to adjust the coordinates of the P state pixels (dark blue dots), effectively altering the visual appearance of the image. During the reverse diffusion process, the role of the MeRAM is to produce controlled noise samples, which are used to update the state of each MTJ based on the applied voltage pulses. The noise in this context is reflected as changes in the coordinates of the P state pixels between consecutive steps, simulating the reverse diffusion process. This adjustment guides the image gradually towards a clearer representation by iteratively "subtracting" the noise introduced during the forward diffusion. By leveraging the tunable switching probabilities of the VC-MTJ, we are able to generate noise samples required by the diffusion model.

The obtain the FID score, we first generate 10000 images. Together with the training set, we load the images to a pre-trained Inception v3 model made by Google. For each image, we take the activations from the last pooling layer as the output, which is a vector of 2048×1 as 2048 activation features in this output layer. Thus, for generative images dataset, the size of output matrix O_g is 2048×10000. For training dataset, the size of the output matrix O_t is 2048×16000. Then the FID score is defined as

$$FID = \left\| u_g - u_t \right\|^2 + \mathrm{Tr}(C_g + C_t - 2\sqrt{C_g C_t}), \tag{9}$$

where u_g and u_t are the means of O_g and O_t by features with the size of 2048×1. C_g and C_t are the covariance matrices of O_g and O_t with the size of 2048×2048. The lower the FID score, the higher the quality of generated images.

Reference

1 Coombes, S., beim Graben, P., Potthast, R. & Wright, J. *Neural fields: theory and applications*. (Springer, 2014).

2 Bressloff, P. C. & Webber, M. A. Front propagation in stochastic neural fields. *SIAM Journal on Applied Dynamical Systems* **11**, 708-740 (2012).

3 Gerstner, W. & Kistler, W. M. *Spiking neuron models: Single neurons, populations, plasticity*. (Cambridge university press, 2002).

4 Li, X. *et al.* Enhancement of voltage-controlled magnetic anisotropy through precise control of Mg insertion thickness at CoFeB| MgO interface. *Applied Physics Letters* **110** (2017).

5 Shao, Y. *et al.* Sub-volt switching of nanoscale voltage-controlled perpendicular magnetic tunnel junctions. *Communications Materials* **3**, 87, doi:10.1038/s43246-022-00310-x (2022).

6 Lee, H., Ebrahimi, F., Amiri, P. K. & Wang, K. L. Design of high-throughput and low-power true random number generator utilizing perpendicularly magnetized voltage-controlled magnetic tunnel junction. *AIP Advances* **7**, 055934, doi:10.1063/1.4978320 (2017).

7 Grezes, C. *et al.* Ultra-low switching energy and scaling in electric-field-controlled nanoscale magnetic tunnel junctions with high resistance-area product. *Applied Physics Letters* **108** (2016).

8 Shiota, Y. *et al.* Evaluation of write error rate for voltage-driven dynamic magnetization switching in magnetic tunnel junctions with perpendicular magnetization. *Applied Physics Express* **9**, 013001 (2015).

9 Carpenter, R. *et al.* Offset Field Control for VCMA-MRAM. *IEEE Transactions on Nanotechnology* (2023).

10 Borders, W. A. *et al.* Integer factorization using stochastic magnetic tunnel junctions. *Nature* **573**, 390-393, doi:10.1038/s41586-019-1557-9 (2019).

11 Torrejon, J. *et al.* Neuromorphic computing with nanoscale spintronic oscillators. *Nature* **547**, 428-431, doi:10.1038/nature23011 (2017).

12 Romera, M. *et al.* Vowel recognition with four coupled spin-torque nano-oscillators. *Nature* **563**, 230-234, doi:10.1038/s41586-018-0632-y (2018).

13 Fong, X. *et al.* Spin-Transfer Torque Memories: Devices, Circuits, and Systems. *Proceedings of the IEEE* **104**, 1449-1488, doi:10.1109/JPROC.2016.2521712 (2016).

14 Liu, S. *et al.* Random Bitstream Generation Using Voltage-Controlled Magnetic Anisotropy and Spin Orbit Torque Magnetic Tunnel Junctions. *IEEE Journal on Exploratory Solid-State Computational Devices and Circuits* **8**, 194-202 (2022).

15 Kang, W., Chang, L., Zhang, Y. & Zhao, W. in *Design, Automation & Test in Europe Conference & Exhibition (DATE), 2017.* 542-547 (IEEE).

16 Zhang, W. *et al.* Analog-type resistive switching devices for neuromorphic computing. *physica status solidi (RRL)–Rapid Research Letters* **13**, 1900204 (2019).

17 Ho, J., Jain, A. & Abbeel, P. Denoising diffusion probabilistic models. *Advances in Neural Information Processing Systems* **33**, 6840-6851 (2020).

18 Suhail, H. *et al.* in *2023 International Electron Devices Meeting (IEDM).* 1-4 (IEEE).

19 Ishii, Y. *et al.* in *2010 Symposium on VLSI Circuits.* 99-100 (IEEE).

20 Ishii, Y. *et al.* in *2012 IEEE International Solid-State Circuits Conference.* 236-238 (IEEE).

21 Giterman, R., Fish, A., Burg, A. & Teman, A. A 4-transistor nMOS-only logic-compatible gain-cell embedded DRAM with over 1.6-ms retention time at 700 mV in 28-nm FD-SOI. *IEEE Transactions on Circuits and Systems I: Regular Papers* **65**, 1245-1256 (2017).

22 Frankel, B., Sarfati, E., Rossi, D. & Wimer, S. Energy efficiency of opportunistic refreshing for Gain-Cell Embedded DRAM. *IEEE Transactions on Circuits and Systems I: Regular Papers* **70**, 1605-1612 (2022).
23 Trentzsch, M. *et al.* in *2016 IEEE International Electron Devices Meeting (IEDM).* 11.15. 11-11.15. 14 (IEEE).
24 Dünkel, S. *et al.* in *2017 IEEE International Electron Devices Meeting (IEDM).* 19.17. 11-19.17. 14 (IEEE).
25 Wan, W. *et al.* A compute-in-memory chip based on resistive random-access memory. *Nature* **608**, 504-512 (2022).
26 Sassine, G. *et al.* in *2018 IEEE International Reliability Physics Symposium (IRPS).* P-MY. 2-1-P-MY. 2-5 (IEEE).
27 Pan, C. & Naeemi, A. Nonvolatile spintronic memory array performance benchmarking based on three-terminal memory cell. *IEEE Journal on Exploratory Solid-State Computational Devices and Circuits* **3**, 10-17 (2017).
28 Jan, G. *et al.* in *2018 IEEE Symposium on VLSI Technology.* 65-66 (IEEE).
29 Dong, Q. *et al.* in *2018 IEEE International Solid-State Circuits Conference-(ISSCC).* 480-482 (IEEE).
30 Garello, K. *et al.* in *2019 Symposium on VLSI Circuits.* T194-T195 (IEEE).
31 Natsui, M. *et al.* Dual-port SOT-MRAM achieving 90-MHz read and 60-MHz write operations under field-assistance-free condition. *IEEE Journal of Solid-State Circuits* **56**, 1116-1128 (2020).
32 Cai, K. *et al.* in *2022 International Electron Devices Meeting (IEDM).* 36.32. 31-36.32. 34 (IEEE).

Supplementary Figures

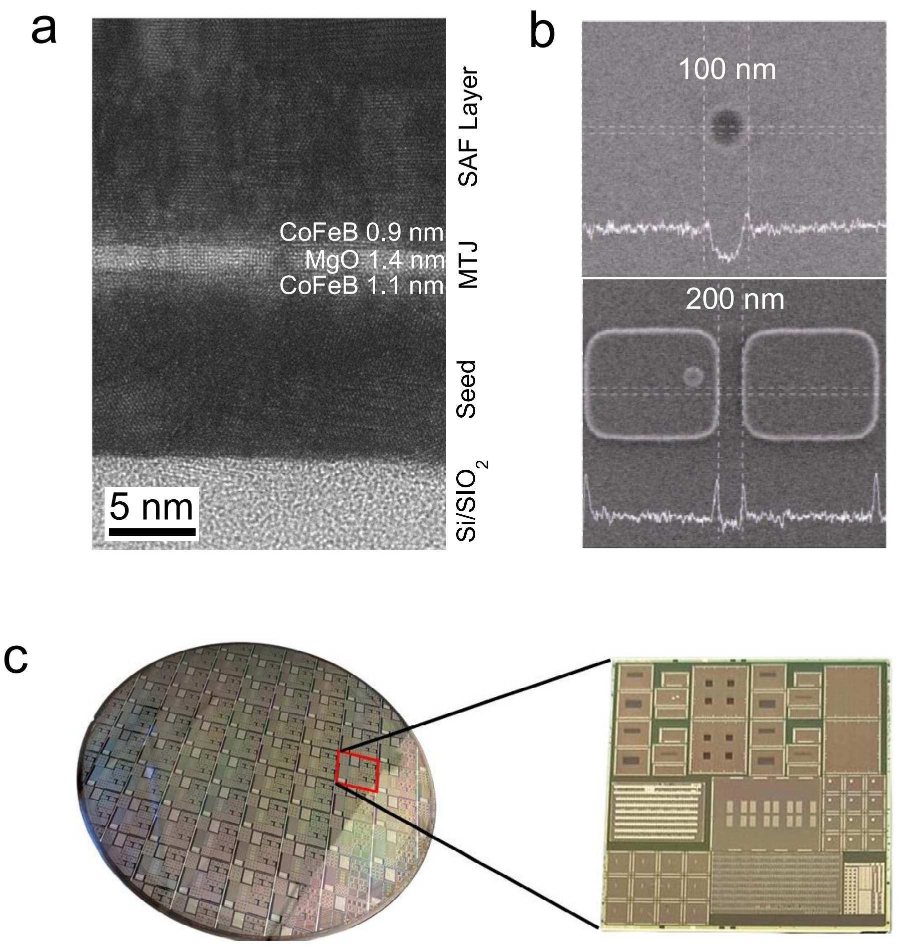

Supplementary Figure 1. Fabrication and characterization of MTJ. a, Transmission electron microscopy (TEM) image of MTJ full stack. **b,** Scanning electron microscope (SEM) image of (up) single MTJ with diameter of 100 nm; (down) MTJ with electrodes. The distance between electrodes is 200 nm. **c,** Wafer-level assembly of MeRAM with surrogating die size 20 X 20 mm.

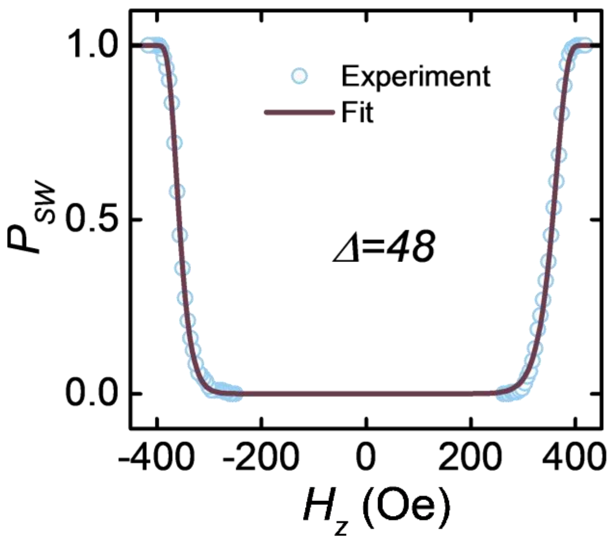

Supplementary Figure 2. Fitting of thermal stability. Thermal stability measurement of a MTJ device by sweeping out-of-plane magnetic field.

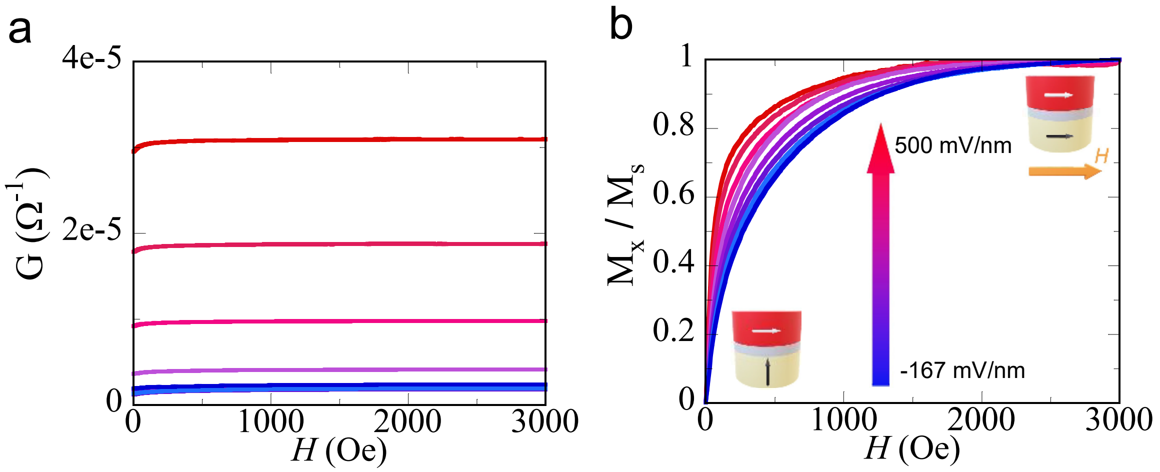

Supplementary Figure 3. Determine VCMA coefficient. a, In-plane field dependence of tunneling conductance for the half-MTJ under different electric fields (gate voltages). **b,** M-H curve after converting the tunneling conductance G to M_x.

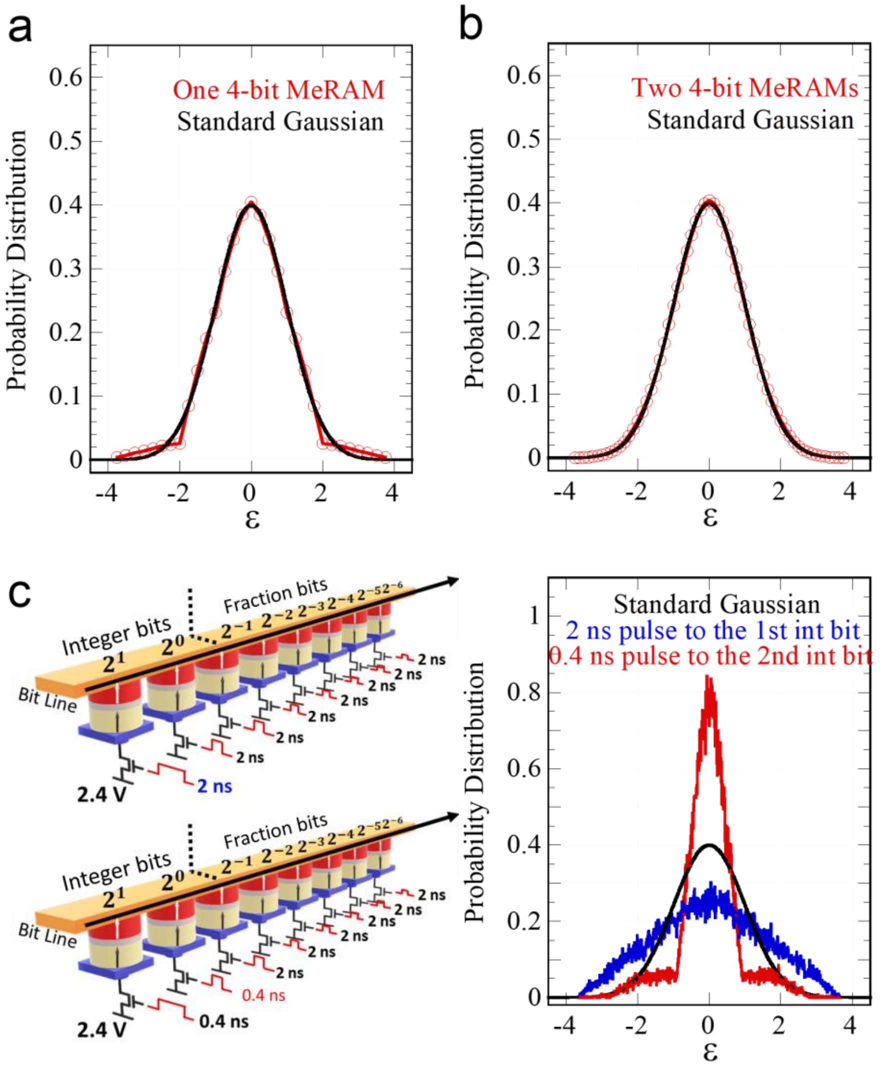

Supplementary Figure 4. More accurate noise distribution. a, Noise distribution of one 4-bit MeRAM unit **b,** Noise distribution of two 4-bit MeRAM units, which is closer to the targeted normal Gaussian distribution. **c**, Unsuitable combinations of gate voltages and pulse widths of 8-bit MeRAM unit result in non-ideal noise distributions.

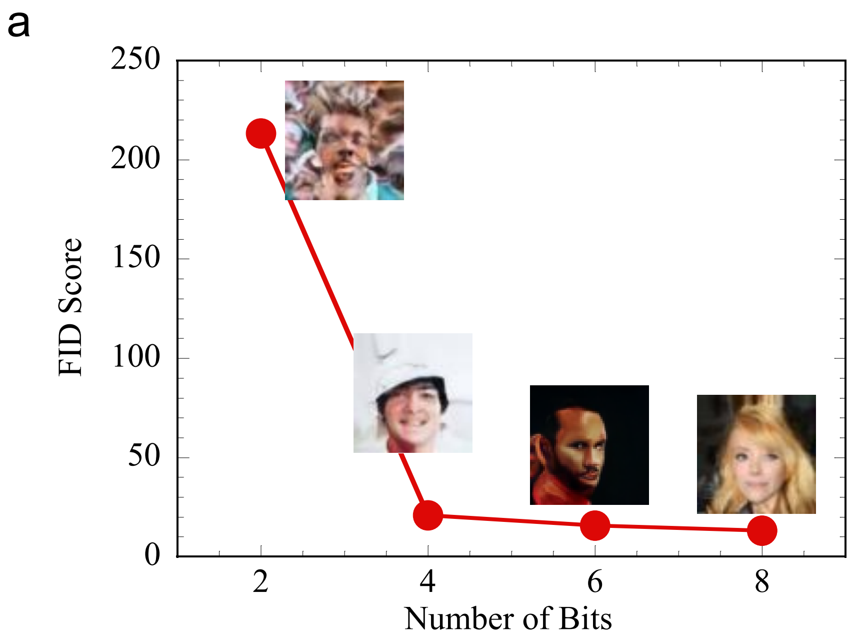

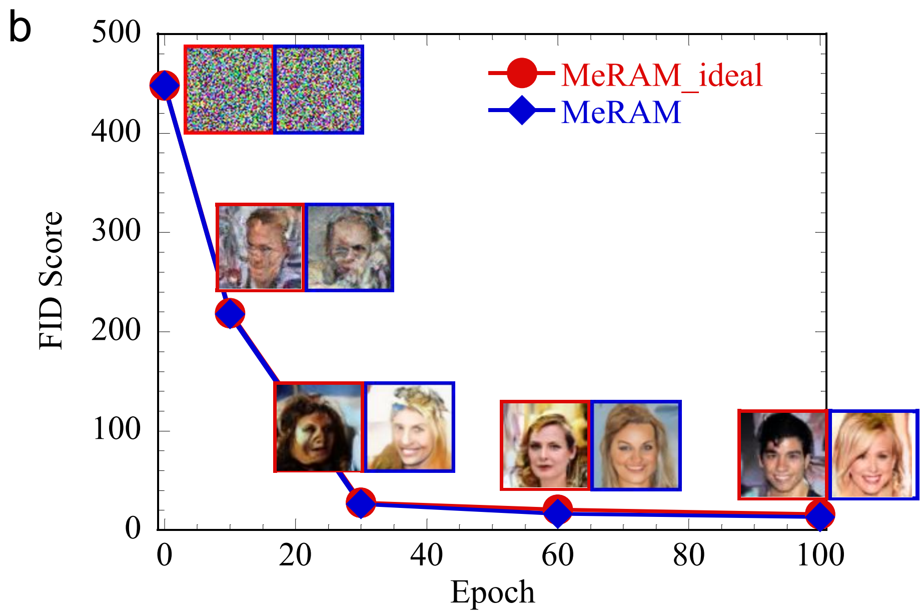

Supplementary Figure 5. Impact of bit precision and device variability on the image generation quality using MeRAM-based diffusion. a, FID score as a function of the number of bits in the MeRAM array, ranging from 2 to 8 bits. The FID score stabilizes beyond 6 bits, and using 4-bit precision already produces relatively good image quality. **b,** Comparison of FID scores

for MeRAM-based diffusion with and without device variability. The "ideal" simulation assumes that the switching probability exactly matches the theoretical model, with every MTJ exhibiting identical behavior and no variance. The results show no significant change in FID score, suggesting that the variability of MTJ switching probabilities across devices does not degrade the image generation performance. The reason for this robustness is that the image generation process in DDPM relies on many small, random adjustments rather than a single precise change. Each step of the process involves adding and removing small amounts of noise repeatedly, which helps the model "average out" any small errors. In other words, because the model uses many steps and combines multiple noise samples, any minor inaccuracies or variations in the noise do not have a large impact on the final image quality. This flexibility allows the system to still perform well even if the switching probabilities of our MTJs are not perfectly aligned with the theoretical predictions.

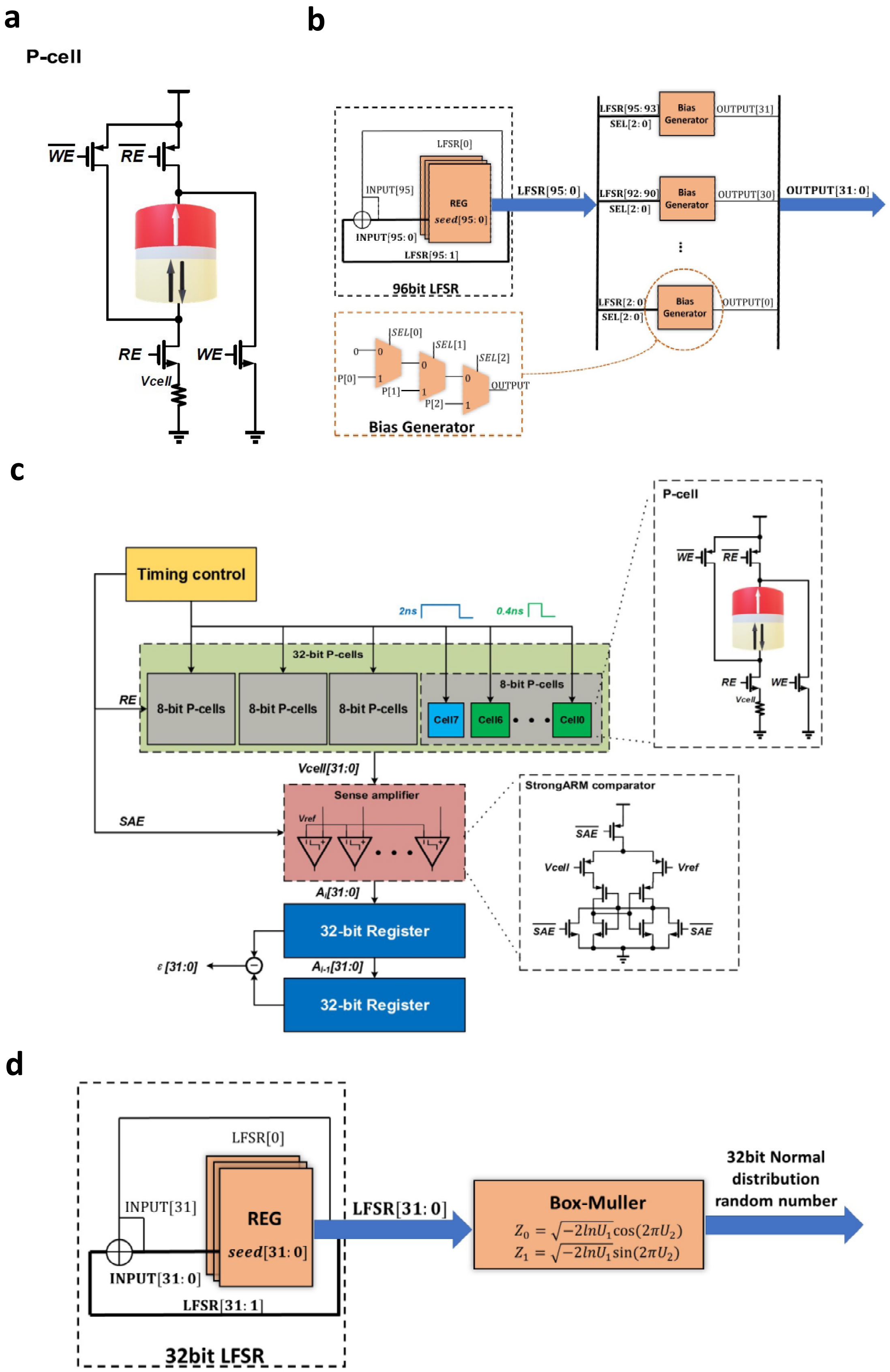
a
P-cell
WE
RE
RE
WE
Vcell
b
LFSR[0]
INPUT[95]
REG
seed[95: 0]
INPUT[95: 0]
LFSR[95: 1]
96bit LFSR
LFSR[95: 0]
LFSR[95: 93]
SEL[2: 0]
Bias Generator
OUTPUT[31]
LFSR[92: 90]
SEL[2: 0]
Bias Generator
OUTPUT[30]
OUTPUT[31: 0]
LFSR[2: 0]
SEL[2: 0]
Bias Generator
OUTPUT[0]
SEL[0]
SEL[1]
SEL[2]
P[0]
P[1]
P[2]
OUTPUT
Bias Generator
c
Timing control
2ns
0.4ns
32-bit P-cells
RE
8-bit P-cells
8-bit P-cells
8-bit P-cells
8-bit P-cells
Cell7
Cell6
Cell0
P-cell
WE
RE
RE
WE
Vcell
Vcell[31:0]
Sense amplifier
Vref
SAE
StrongARM comparator
SAE
Vcell
Vref
SAE
SAE
Ai[31:0]
32-bit Register
ε [31:0]
Ai-1[31:0]
32-bit Register
d
LFSR[0]
INPUT[31]
REG
seed[31: 0]
INPUT[31: 0]
LFSR[31: 1]
32bit LFSR
LFSR[31: 0]
Box-Muller
$Z_0 = \sqrt{-2lnU_1}\cos(2\pi U_2)$
$Z_1 = \sqrt{-2lnU_1}\sin(2\pi U_2)$
32bit Normal distribution random number

Supplementary Figure 6. Compared to CMOS-based technology. a, P-cell as basic unit of MeRAM unit. A P-cell includes 4 transistors to separate the read and write operation. **b,** The CMOS-based biased random bit generator includes a 96-bit linear-feedback shift register (LFSR) pseudo random number generator (PRNG) and a bias generation circuit. **c,** 32-bit VC-MTJ unit consists of P-cells, timing control, readout sense amplifiers, and a subtraction circuit. **d,** The reference CMOS-based normal random number generator includes a 32-bit LFSR-based PRNG and a Box-Muller transform block.

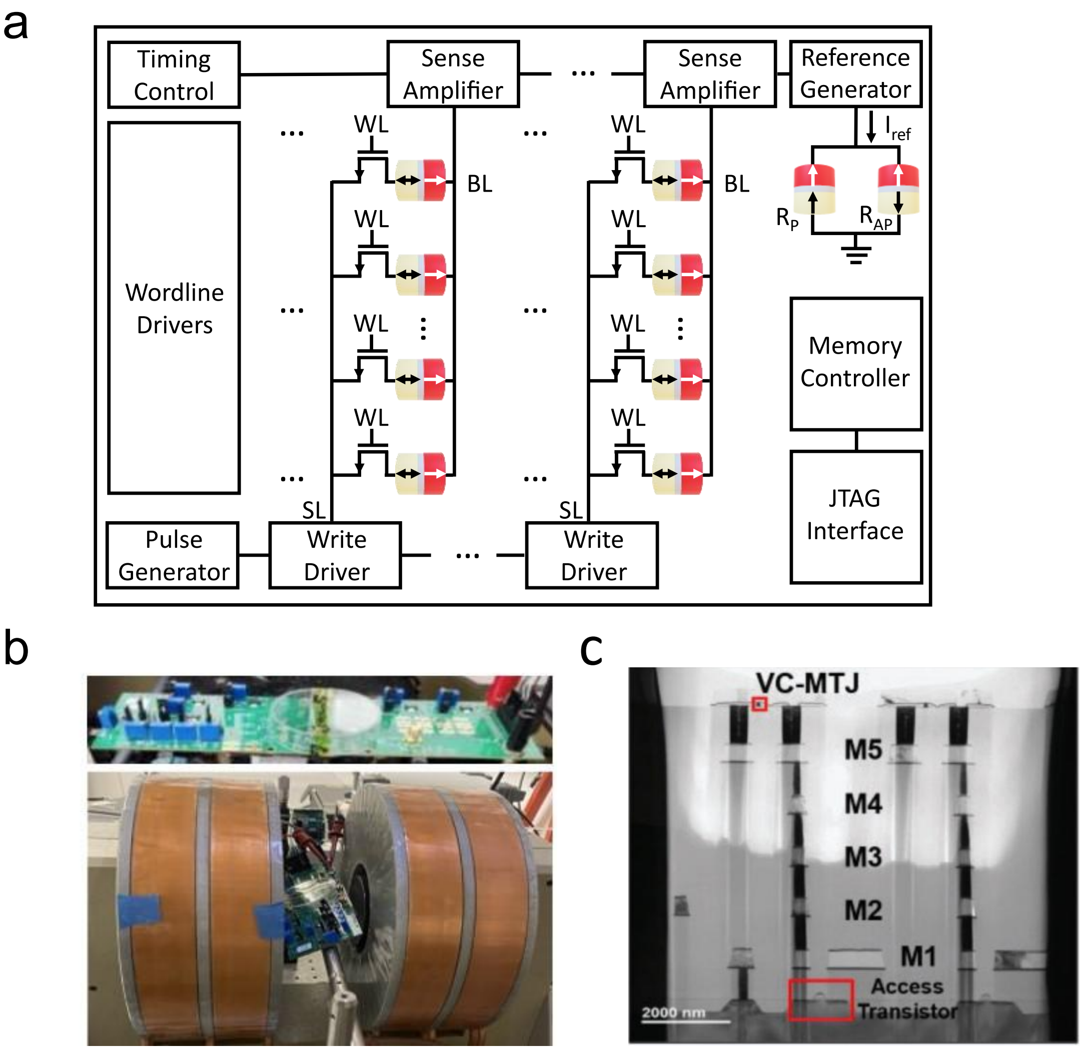

Supplementary Figure 7. An 80-by-80 MeRAM array is integrated BEOL (Back End of Line) on a CMOS circuit chip. a, Block diagram of the MeRAM circuit. The MTJs are arranged in an 80-by-80 pattern, and each column has a write driver and a read sense amplifier. All sense amplifiers share a common reference. The timing control generates control signals that transition faster than the digital clock. The chip communicates with a JTAG interface. **b,** Test PCB is situated in a magnet and connected to a microcontroller unit (MCU). **c,** TEM image of 1T (Transistor)-1MTJ cell[18].

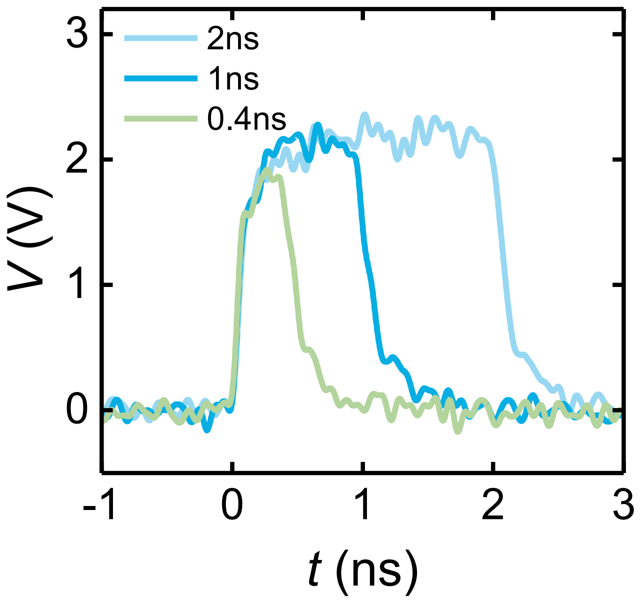

Supplementary Figure 8. Nanosecond voltage pulse shape calibration. Voltage pulses of 2.1 V captured by oscilloscope.

Supplementary Tables

Biased RBG	Energy (fJ)	Area (μm^2)	Throughput (Mbps)	FOM
P-cell	38-65	6.3	125	16-28
LFSR + Bias generator	234	29.9	129	1

Supplementary Table 1. Performance of a single P-cell compared to LFSR + Bias generator in generating biased RBG.

32-bit Normal RNG	Energy (pJ)	Area (μm^2)	Throughput (Mbps)	FOM
MeRAM	3.4	983	91	5309
LFSR + Box-Muller	104.9	10953	6	1

Supplementary Table 2. Performance of MeRAM compared to LFSR + Box-Muller in generating 32-bit normal RNG.

	SRAM [19,20]	4T GC-eDRAM [21,22]	MeRAM	FeFET [23,24]	RRAM [25,26]	STT-MRAM[27-29]	SOT-MRAM [27,30-32]

Cell Size (F^2)	415	293	16	50	100	16	24
Write Energy (fJ/bit)	0.24	0.33	5	5	10^2-10^5	120	220
Write Speed (ns)	1.4	0.6	0.5	10	100	10-20	0.5
Non-volatility	No	No	Yes	Yes	Yes	Yes	Yes
Retention Power (pW/bit)	~9.3	~7.2	0	0	0	0	0
Endurance	Infinite	Infinite	> 10^{11}	>10^4	>10^6	>10^{10}	>10^{12}

Supplementary Table 3. Performance of MeRAM compared with other volatile and non-volatile memories.

Parameter	Value
Magnetization M_s [A/m]	10^6
Uniaxial anisotropy K_u [$\mathrm{J \cdot m^{-3}}$]	7.114×10^5
Damping constant α	0.1
VCAM coefficient ξ [$\mathrm{fJ/V \cdot m}$]	44
MgO thickness t [nm]	1.8
Free layer thickness t_{FM} [nm]	0.95
In-plane magnetic field H_x [Oe]	350
Critical dimension CD [nm]	100

Supplementary Table 4. Simulation parameters for the macrospin model.
